# Establishment of a Flow Cytometry Protocol for Binarily Detecting Circulating Tumor Cells with EGFR Mutation

**DOI:** 10.3390/diseases13120406

**Published:** 2025-12-17

**Authors:** Cheng-Yu Chang, Chia-Chun Tu, Shian-Ren Lin, Chih-Hao Fang, Po-Wei Tseng, Wan-En Liao, Li-Yun Huang, Shiu-Lan Wang, Wan-Yu Lai, Yee Chao, Yen-Ling Chiu, Jan-Mou Lee

**Affiliations:** 1 Division of Chest Medicine, Department of Internal Medicine, Far Eastern Memorial Hospital, New Taipei City 220, Taiwan; koala2716@yahoo.com.tw; 2 Department of Electrical Engineering, Yuan Ze University, Taoyuan City 320, Taiwan; 3 FullHope Biomedical Co., Ltd., New Taipei City 241405, Taiwan; garytu@fhb.com.tw (C.-C.T.); jimmylin@fhb.com.tw (S.-R.L.); jackfang@fhb.com.tw (C.-H.F.); petertseng@fhb.com.tw (P.-W.T.); erinliao@fhb.com.tw (W.-E.L.); doryhuang@fhb.com.tw (L.-Y.H.); bellewang@fhb.com.tw (S.-L.W.); wendylai@fhb.com.tw (W.-Y.L.); ychao1957@gmail.com (Y.C.); 4 Central Clinic and Hospital, Taipei City 106441, Taiwan; 5 Division of Gastroenterology and Hepatology, Department of Medicine, Taipei Veterans General Hospital, Taipei City 112201, Taiwan; 6 Division of Nephrology, Department of Medicine, Far Eastern Memorial Hospital, New Taipei City 220, Taiwan; 7 Department of Medical Research, Far Eastern Memorial Hospital, New Taipei City 220, Taiwan; 8 Graduate Institute of Medicine and Graduate Program in Biomedical Informatics, Yuan Ze University, Taoyuan City 320, Taiwan; 9 Graduate Institute of Clinical Medicine, National Taiwan University College of Medicine, Taipei City 100, Taiwan

**Keywords:** circulating tumor cell, non-small-cell lung cancer, epidermal growth factor, targeted therapy, flow cytometry

## Abstract

Background: Patients with EGFR-mutated non-small cell lung cancer (NSCLC) respond well to EGFR tyrosine kinase inhibitors (TKIs), but current EGFR mutation profiling relies on invasive tumor biopsies. Developing less invasive approaches, particularly proteomic evaluation of circulating tumor cells (CTCs) for EGFR mutation profiling, remains crucial. Methods: A flow cytometry method for detecting EGFR^L858R^-bearing CTCs was established by spiking NCI-H1975 cells into blood from cancer-naive donors. The method was then applied to blood samples from 21 NSCLC patients and 10 cancer-naive donors. Results: The gating strategy was defined by CD45^−^CK-7/8^+^CK-14/15/16/19^−^EpCAM^+^vimentin^+^EGFR^L858R^, with a cut-off value of 5 cells/mL. The method yielded positive results in all seven patients with the EGFR^L858R^ mutation and negative results in all ten cancer-naive donors. Compared to the PCR-based reference method, the approach showed 100% positive and 71% negative agreement. Crucially, our in-house method detected EGFR^L858R^-bearing CTCs in three patients initially identified as EGFR wild-type and one patient with a different EGFR mutation. The remaining samples were concordant with PCR. Notably, two patients with these discordant results received EGFR-TKIs and experienced partial responses. Conclusions: This study introduces a feasible, less invasive proteomic approach for binarily detecting EGFR mutations in CTCs, offering a novel means for patient identification.

## Simple Summary

This study conceptualized a method using flow cytometry to assess EGFR mutations from peripheral blood, demonstrating high concordance with the mutational profile of primary tumors. This innovative approach provides clinicians with a rapid tool for assessing the applicability of EGFR-targeted therapy. Furthermore, it facilitates continuous monitoring of mutational changes during treatment, enabling precise adjustments to the therapeutic regimen to maximize efficacy while minimizing adverse events.

## 1. Introduction

Epidermal growth factor receptor (EGFR) mutations are prevalent in 38.4% to 49.1% of non-small-cell lung cancer (NSCLC) patients [1,2]. Specific EGFR mutations, including exon 19 deletion, L858R, and T790M [3], significantly enhance tumor sensitivity to EGFR tyrosine kinase inhibitors (EGFR-TKIs). For example, patients with EGFR^T790M^ NSCLC achieve a three-fold higher objective response rate with osimertinib-based therapy [4]. Similarly, NSCLC patients with exon 19 deletion or L858R mutations show superior clinical responses to gefitinib and erlotinib [5]. Consequently, the European Society for Medical Oncology recommends routine EGFR-mutation profiling for NSCLC patients to guide the prescription of selective EGFR-TKIs for those with TKI-sensitive mutations [6].

To profile EGFR mutation, several companion diagnostics (CDx), like Cobas^®^ EGFR mutation test and FoundationOne^®^ Liquid CDx, have been developed [7,8]. These tests primarily rely on molecular techniques, such as polymerase chain reaction (PCR) and next-generation sequencing (NGS), for EGFR mutation analysis in tissue samples [9,10]. While PCR-based CDx are well-established, rapid, and cost-effective for common TKI-sensitive mutations [7,11], and NGS offers accurate profiling for tumor heterogeneity or rare mutations [12], both typically necessitate invasive tumor biopsies [13]. Furthermore, PCR-based EGFR CDx shows limitations, including an estimated 11.3% false-negative rate for solid tissue profiling, up to 80% false-positives for S768I mutation (while detecting exon 20 insertion and exon 19 deletion) [14,15], and an overall analytical agreement of merely 89% [16]. Such inconsistencies can significantly misguide clinical diagnosis and treatment decisions. Despite NGS’s accuracy for TKI-sensitive mutations, its extended turnaround time (more than 7 days) and high cost (typically USD $ 2478) often delay treatment [17]. Therefore, there is a pressing need for more precise, rapid, and less invasive methods to detect EGFR mutations in NSCLC patients.

To overcome the limitations of tissue-based EGFR CDx, peripheral blood-based surrogate markers, such as cell-free DNA (cfDNA) and circulating tumor cells (CTCs), have emerged as promising alternatives for EGFR mutation assessment [18]. CTCs are cells originating from primary tumors that survive in circulation and contribute to metastasis [19]. These cells exhibit phenotypic heterogeneity, commonly classified as epithelial (EpCAM^+^vimentin^−^), mesenchymal (EpCAM^−^vimentin^+^), and hybrid (EpCAM^+^vimentin^+^) based on their marker expression [19]. Given this shared phenotypic heterogeneity with primary tumors, proteomic or transcriptomic analysis of CTCs offers a promising, less invasive approach for guiding targeted therapies and evaluating clinical responses [20].

To characterize CTCs, enrichment techniques (e.g., size exclusion, immune-based selection, or precipitation) are used, followed by genomic/proteomic profiling via molecular-based CDx, mass cytometry, or flow cytometry [21]. However, current CTC enumeration techniques are limited in enriching all CTC types, potentially skewing downstream characterization of CTCs [22]. Specifically, mass cytometry suffers from sample variations, affecting result consistency in routine CTC phenotype analyses [23]. While flow cytometry sensitively detects CTCs in peripheral blood, it traditionally cannot identify EGFR mutations directly on CTCs [24]. Addressing these limitations, our study developed a novel flow cytometry method to directly detect CTCs with EGFR^L858R^ mutations in peripheral blood. We then applied this method to evaluate the performance of detecting EGFR^L858R^-bearing CTCs in NSCLC patients and compared the results with PCR-based EGFR mutation profiling.

## 2. Materials and Methods

### 2.1. Reagents and Antibodies

Table 1 and Table 2 list the reagents and antibodies used in this study. All reagents and antibodies were aliquoted and stored under specific conditions recommended by the manufacturer. Before applying each antibody—except for the isotype control—in immunostaining, optimal dilution factors were determined by performing immunostaining on positive control samples: peripheral blood mononuclear cells for CD45; H1975 cells for EpCAM, EGFR^L858R^, CK-7/8, and vimentin; and SW480 cells for CK-14/15/16/19, using a series of serial dilutions (Supplementary Figure S1).

### 2.2. Laboratory-Based Evaluation of CTCs

#### 2.2.1. Cell Lines

Human NSCLC cell line NCI-H1975 (RRID CVCL_1511) was obtained from the American Type Culture Collection (ATCC, Manassas, VA, USA), and human NSCLC cell line A549 (RRID CVCL_A549) and human breast cancer cell line MCF-7 (RRID CVCL_0031) were obtained from the Bioresource Collection and Research Center (Hsinchu, Taiwan). Cells were maintained in accordance with the suppliers’ instructions. Briefly, NCI-H1975, A549, and MCF-7 cells were cultured in ATCC-modified RPMI 1640 medium (Thermo Fisher Scientific, Waltham, MA, USA), Ham’s F-12K (Kaighn’s) medium (Thermo Fisher Scientific), and Eagle’s minimum essential medium (ATCC), respectively. All media contained 10% (*v*/*v*) fetal bovine serum (Thermo Fisher Scientific), 100 units/mL penicillin G, and 100 μg/mL streptomycin and were refreshed every 2 to 3 days. Cells were detached using TrypLE select (Thermo Fisher Scientific) once they reached 80% confluence for further experiments. All experiments were conducted within 10 passages to ensure cell consistency.

#### 2.2.2. Immunostaining

Immunostaining was performed in accordance with our previously described protocol [25]. Briefly, detached cancer cells were incubated with antibodies targeting specific surface markers in a cell staining buffer (BioLegend, San Diego, CA, USA) for 30 min. Subsequently, the cells were washed with a staining buffer, fixed, and permeabilized using a Foxp3 transcription factor staining buffer set (eBioscience, San Diego, CA, USA), followed by staining with additional antibodies targeting internal markers for 30 min. Next, the stained cells were analyzed using a flow cytometer (Navios; Beckman Coulter, Brea, CA, USA). Finally, the gating of each marker was set through the fluorescence profile of the corresponding unstained control sample or fluorescence minus one control (Supplementary Figure S2).

#### 2.2.3. Tumor Cell Spike-In Assay

Briefly, 10 mL of anticoagulated peripheral blood from a cancer-naïve donor was aliquoted into 1 mL portions. Then, detached NCI-H1975 cells were spiked into peripheral blood at concentrations of 2, 5, 10, and 25 cells/mL, mimicking CTC-containing blood samples. Finally, the blood samples were undergoing erythrolysis with an RBC lysis buffer (BioLegend), followed by immunostaining for the remaining cells and a fluorescence profile analysis via flow cytometer. All sample preparation steps were finished within 28 h after initiation.

### 2.3. Clinical Evaluation of CTCs

#### Ethical Considerations and Analysis of CTCs

This study was conducted in accordance with the Declaration of Helsinki. The study protocol was approved by the Institutional Review Board of Far Eastern Memorial Hospital, Taiwan (approval no. 108170-E, approved in February 2020).

Patients with NSCLC and cancer-naïve donors who visited Far Eastern Memorial Hospital from March 2021 to July 2024 were invited to participate in this study. Patients meeting the following criteria were included for analysis: being above 20 years of age, weighing more than 50 kg, having an established diagnosis of NSCLC as per clinical practice, available EGFR mutation test results (PCR), having no human immunodeficiency virus or *Treponema pallidum* infection, receiving no ongoing autoimmune disease treatment, and having no history of chemotherapy or radiotherapy within 1 month before blood collection. Patients with severe cardiovascular, hepatic, and renal disorders; severe or uncontrolled infection; or social disorders were excluded. The inclusion and exclusion criteria for cancer-naïve participants (CNPs) were identical to those of patients with NSCLC, with the exception of a cancer diagnosis. After the patients signed an informed consent form, 20 mL of peripheral blood was collected, aliquoted into 1 mL portions, and subjected to erythrolysis, immunostaining, and fluorescent profiling on a flow cytometer. All sample preparation steps were finished within 28 h after blood collection.

### 2.4. Data Acquisition and Statistical Analysis

Fluorescence data obtained from flow cytometry were acquired using Kaluza Analysis Software version 2.3 (Beckman Coulter) and presented in scatter plots for CTC gating. Gated CTCs were counted, summarized as mean ± standard error of the mean, and plotted on bar charts or scatter plots by using GraphPad Prism software version 9.5.1 (GraphPad Software, Boston, MA, USA). Fluorescence profiles from all stained cells (approximately 2.5 × 10^5^ cells/sample) were acquired. The statistical significance of CTC counts between patients with NSCLC and cancer-naïve donors was evaluated using one-way analysis of variance (ANOVA) with Dunnett’s test for post hoc analysis. In the statistical analysis results, bars representing statistically different volumes (*p* < 0.01) were labeled with asterisks (**).

## 3. Results

### 3.1. Establishment of a Pedigree to Identify EGFR^L858R^-Bearing CTCs in Peripheral Blood Through Flow Cytometry

**Figure 1:**
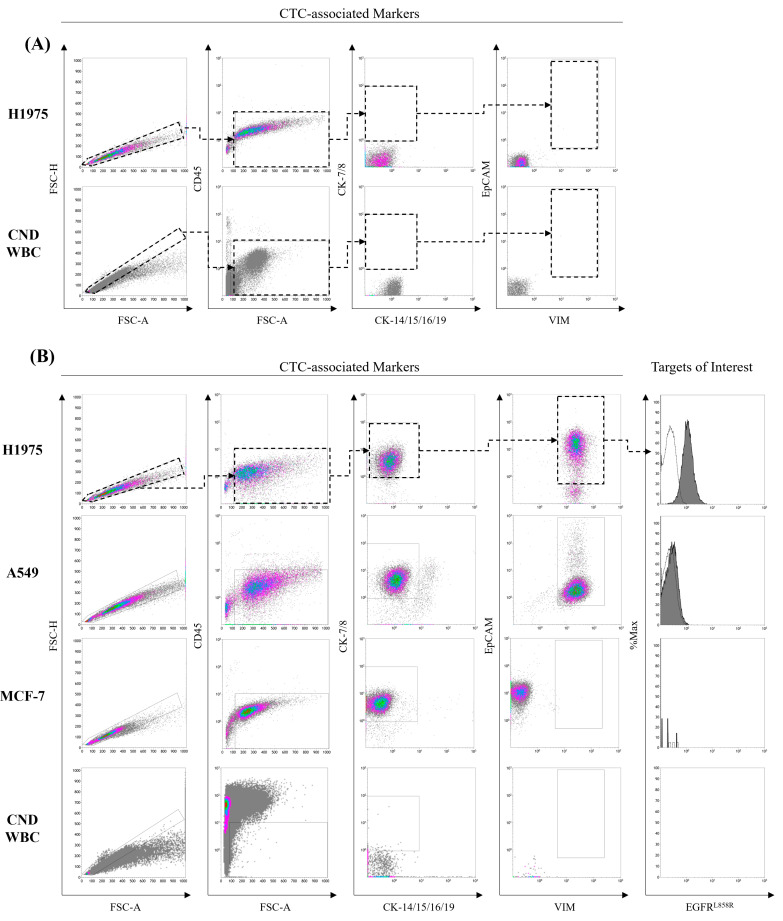
Establishment of gating for identifying CTCs and cells with EGFR^L858R^ mutations through NSCLC cell lines. The detailed procedure is described in the Materials and Methods section. Briefly, NCI-H1975 (EGFR^L858R^), A549 (EGFR wild type), and MCF-7 (breast cancer cells, negative control) cells were detached and stained with specific antibody cocktails, followed by flow cytometry analysis. After the data were collected, signals from tumor cells were sequentially filtered using CD45, CK-7/8, CK-14/15/16/19, EpCAM, and vimentin to mimic the signals of CTCs (black dashed arrow and dashed box). To evaluate the specificity of signaling from EGFR^L858R^ mutations, fluorescence from an EGFR^L858R^-specific antibody was measured, coupled with its isotype control. To exclude interference from normal cells in peripheral blood, a peripheral blood sample was collected from a cancer-naïve donor and subjected to immunostaining and flow cytometry analysis. Gating of all markers was set based on the (**A**) unstained control sample and (**B**) stained samples. CND, cancer-naïve donors.

We first established a flow cytometry protocol to identify circulating tumor cells (CTCs) in peripheral blood. Current CTC identification methods typically rely on markers such as CD45 (leukocyte marker), cytokeratin (CK-7/8, CK-14/15/16/19), EpCAM (epithelial cell marker), and vimentin (mesenchymal cell marker) [26]. To define the appropriate CTC identification gate for our study, we examined the expression of these markers on several cancer cell lines: NCI-H1975 (with EGFR^L858R^ mutation) [27], A549 cells (with wild-type EGFR) [28], and MCF-7 cells (low expression of EGFR) [29]. Via comparing fluorescence profile with unstained control samples (Figure 1A), all tested cell lines were negative for CD45 and CK-14/15/16/19, but positive for CK-7/8 (Figure 1B). EpCAM and vimentin expression revealed distinct patterns: NCI-H1975 and A549 cells expressed both EpCAM and vimentin, while MCF-7 cells expressed EpCAM alone (Figure 1B). Given the reported rarity of purely epithelial-like CTCs in NSCLC patients [30,31,32], we defined our CTC gate as CD45^−^CK-7/8^+^CK-14/15/16/19^−^EpCAM^+^vimentin^+^.

After establishing the CTC gating, we proceeded to establish the gating for EGFR^L858R^. We stained NCI-H1975 (EGFR^L858R^) and A549 (EGFR^WT^) cells with an EGFR^L858R^-specific antibody and analyzed their fluorescence using flow cytometry. MCF-7 cells, which have low EGFR expression [26], served as our negative reference for EGFR in this analysis. To account for nonspecific binding, we also included isotype controls with an identical fluorophore. As shown in Figure 1, the fluorescence intensity of NCI-H1975 cells was significantly higher than that of A549, MCF-7, and the isotype control. Conversely, the fluorescence patterns of A549 and MCF-7 cells overlapped with the isotype control. These results confirm that the EGFR^L858R^-specific antibody successfully identified EGFR^L858R^ mutations in tumor cells. Based on the isotype control’s fluorescence, we then set the gating for EGFR^L858R^. In subsequent experiments, we further examined the accuracy of this CTC detection method in identifying cancer cells with EGFR^L858R^ mutations from peripheral blood.

**Figure 2:**
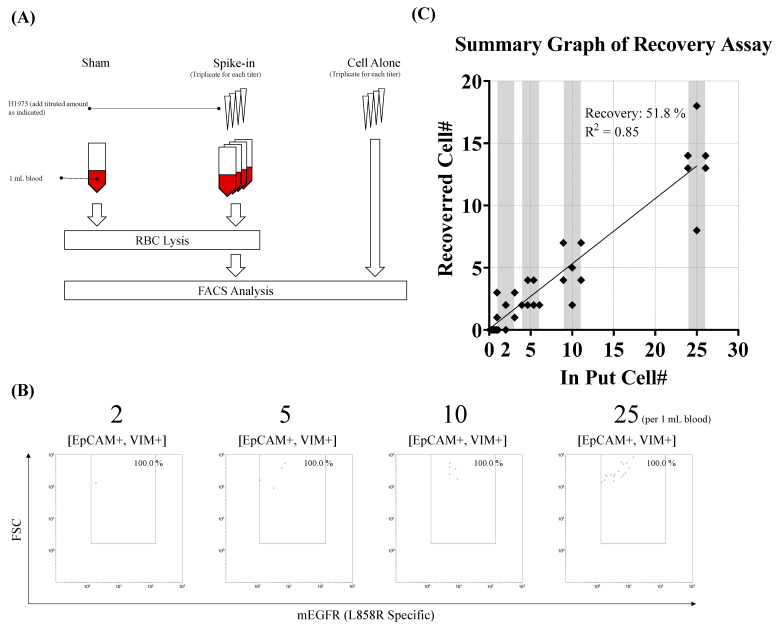
Detection of EGFR^L858R^-bearing tumor cells spiked in peripheral blood samples through the proposed method. To determine whether our method can specifically detect EGFR^L858R^-bearing tumor cells in peripheral blood, we conducted a spike-in assay. (**A**) Spike-in assay. Briefly, NCI-H1975 cells were spiked into 1 mL of peripheral blood from a cancer-naïve donor at concentrations ranging from 2 to 25 cells/mL, followed by erythrolysis, immunostaining, and flow cytometry analysis, as described in the Materials and Methods section. After data were collected, tumor cells were filtered using the gating strategy described in Figure 1. (**B**) Representative example. (**C**) X-Y scatter plot comparing the numbers of detected and spiked tumor cells.

To determine whether our in-house method could detect EGFR^L858R^-bearing CTCs from a blood sample, we spiked NCI-H1975 cells into peripheral blood samples collected from a cancer-naïve donor at concentrations of 2, 5, 10, and 25 cells/mL, then counted the events within the EGFR^L858R^/forward scatter gate as previously described (Figure 2A). As shown in Figure 2B, all detected events fell within the EGFR^L858R^ positive gating area across all tested concentrations. This confirmed that our in-house method successfully detected EGFR^L858R^-bearing CTCs from peripheral blood. However, quantification accuracy showed limitations. At each concentration, the average CTC counts were 1.6 (for 2 cells/mL spiked), 3.1 (for 5 cells/mL), 5.2 (for 10 cells/mL), and 13.5 cells (for 25 cells/mL), with an average recovery rate of 51.8% (Figure 2C). The observed variability in the recovery rate indicates that our in-house method cannot accurately quantify EGFR^L858R^-bearing CTCs. Nevertheless, by establishing a cut-off at 5 cells/mL—due to the pseudo-negative results identified at the 2 cells/mL concentration—the method can be used to binarily detect these CTCs in peripheral blood. In summary, we developed a flow cytometry-based method capable of specifically detecting EGFR^L858R^-bearing CTCs with a cut-off value of 5 cells/mL.

### 3.2. Detection of EGFR^L858R^-Bearing CTCs in Blood Samples from Patients with NSCLC

To assess the real-world applicability of our in-house method, a cohort of 21 patients with NSCLC (NSCLC group) and 10 cancer-naïve donors (CNP group) were enrolled from Far Eastern Memorial Hospital between March 2021 and July 2024. Table 3 summarizes the demographics of the participants. The male-to-female ratio was 1:1.8 in the NSCLC group and 1.5:1 in the CNP group. The median age was similar between the two groups (65.5 vs. 61.5 years, *p* = 0.06). At enrollment, one patient received a diagnosis of stage IIIb NSCLC, and the remaining patients received a diagnosis of stage IV NSCLC. EGFR mutation profiling performed using the Cobas EGFR Mutation Test v2 revealed that eight patients carried wild-type EGFR, seven patients carried EGFR^L858R^ mutations, and the remaining patients carried other types of mutations (e.g., G719X, exon 19 deletion, exon 20 deletion, and S768I). As NGS was not covered by National Health Insurance in Taiwan during the study period, no NGS results were available for the participants in the NSCLC group at enrollment.

**Figure 3:**
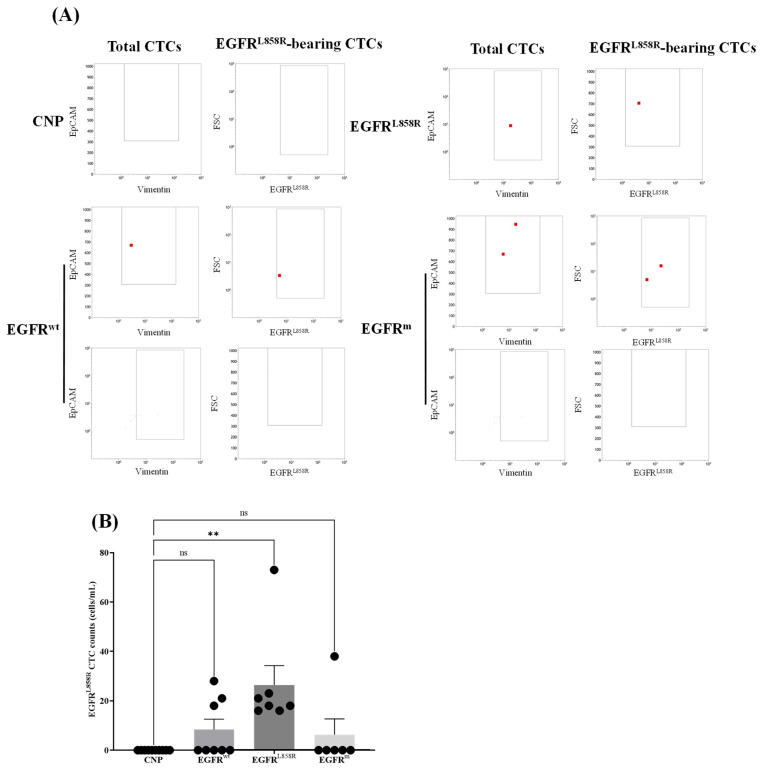
Validation of the proposed CTC detection method with actual samples. Peripheral blood samples collected from patients with NSCLC and healthy controls were analyzed using the pedigree applied in the NCI-H1975 recovery assay. CTCs were identified by the following markers: CD45^−^, CK-7/8^+^, EpCAM^+^, VIM^+^, and EGFR^L858R^. Panel (**A**) displays representative images of total and EGFR^L858R^-bearing CTCs across all groups, while Panel (**B**) summarizes the group data. Results are presented as mean ± standard error of the mean (SEM), with individual data points indicated. Statistical analysis was performed using one-way ANOVA with Dunnett’s post-hoc test. Statistically significant column pairs (*p* < 0.01) are denoted with **. EGFR^wt^, wild-type EGFR; EGFR^m^, mutant EGFR other than L858R; EGFR^L858R^, EGFR with L858R mutation.

When investigating EGFR^L858R^-mutant CTCs with our in-house method, all 10 samples from the CNP group tested negative. The method successfully identified all 7 patients with the EGFR^L858R^ mutation (concentrations: 16–75 cells/mL) as positive, while also detecting positive results in 3 of 8 patients with wild-type EGFR (18–28 cells/mL) and 1 of 4 patients with other EGFR mutations (38 cells/mL). The remaining 8 patients, consisting of 5 with wild-type EGFR and 3 with other EGFR mutations, showed negative results (Figure 3B). In comparison to the Cobas EGFR Mutation Test, our in-house method produced 4 discordant results, demonstrating overall, positive, and negative percentage agreements of 81%, 100%, and 71%, respectively (Table 4). Consistent with guidelines recommending EGFR-TKIs solely for patients with EGFR-mutated NSCLC [33], 12 patients with EGFR-mutant tumors were scheduled to receive EGFR-TKI therapy (11 with open-labeled treatment and 1 with closed-labeled treatment). At the time of writing, 2 of 12 patients had received EGFR-TKI therapy and had an observable clinical response. Their treatment detail would be described in the following section.

### 3.3. Case Presentation 1

**Figure 4:**
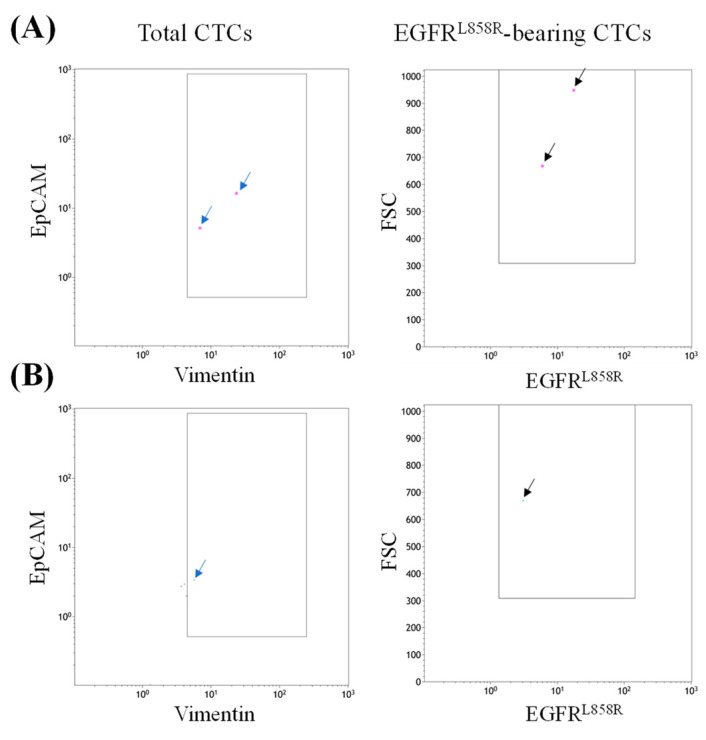
EGFR^L858R^-bearing CTCs detection in the representative cases. Panels (**A**,**B**) present the detection results from two participants who exhibited conflicting results between the PCR assay and our in-house method, both of whom received EGFR-targeted therapies. Detected CTCs are indicated with blue arrows, and EGFR^L858R^-bearing CTCs with black arrows.

This patient was initially diagnosed with stage IVa (T4N0M1b) lung cancer in the left lower lobe. The patient underwent video-assisted lobectomy and lymphadenectomy, removing the left lung lobe and lymph nodes at stations 7 to 9. Subsequent pathological findings updated the TNM stage to T3N2M1b. PCR-based EGFR mutation profiling revealed the tumor harbored the EGFR^Δ19del^ mutation, leading to treatment with afatinib from August 2023 until March 2025, when therapy was discontinued due to grade I–II rash and grade III paronychia. During treatment, the patient achieved partial remission according to the Response Evaluation Criteria in Solid Tumors (RECIST) version 1.1. In April 2023, the patient enrolled in our trial and underwent testing using our in-house method, which detected EGFR^L858R^-bearing CTCs (Figure 4A). Given the partial remission observed with afatinib, the patient’s therapy was switched to osimertinib in April 2025. Following this change, the patient again experienced partial remission according to RECIST version 1.1. At the time of this report, the patient’s disease remains stable, with no significant adverse events reported.

### 3.4. Case 2 Presentation

This patient was diagnosed with lung adenocarcinoma with cerebral metastases. PCR-based EGFR mutation profiling indicated that the tumor harbored wild-type EGFR. The patient received five cycles of etoposide combined with cisplatin from April 2024 to October 2024 due to progressive disease, as assessed by RECIST version 1.1. In May 2024, the patient participated in our trial, and our in-house method identified EGFR^L858R^-bearing CTCs in the patient’s peripheral blood (Figure 4B). Based on this finding, the patient received off-label erlotinib from October 2024 to March 2025, during which partial remission was observed. Unfortunately, progressive disease was detected in March 2025, and the patient passed away in May 2025.

## 4. Discussion

This study conceptually proposes a novel method for investigating EGFR mutation from peripheral blood by detecting EGFR^L858R^-bearing CTC using flow cytometry. This method involves erythrolysis followed by antibody staining targeting CD45, CK-7/8, CK-14/15/16/19, EpCAM, and vimentin to define CTCs, as well as EGFR^L858R^-specific antibody staining to evaluate the expression of EGFR^L858R^ in CTCs. Overall, this method can specifically and binarily detect EGFR^L858R^-bearing CTCs with a cut-off value of 5 cells/mL. We applied this in-house method to detect EGFR^L858R^-bearing CTCs in peripheral blood samples from NSCLC patients and CNPs. All CNPs and patients with EGFR^L858R^ mutant NSCLC were detected correctly, demonstrating a 100% positive percentage agreement with PCR-based EGFR mutation profiling. Notably, our method also detected positive results in samples from three patients with wild-type NSCLC and one with NSCLC carrying another mutation, resulting in a 71% negative percentage agreement with PCR profiling. Following treatment with EGFR-TKIs, two patients with these discordant results from PCR analysis experienced partial remission. To the best of our knowledge, this is the first method to detect EGFR mutations in CTCs through proteomics.

To reduce invasiveness, cfDNA and CTCs have been proposed for profiling EGFR mutations, leading to the FDA approval of corresponding CDx in the U.S [18]. However, comparisons of EGFR mutation profiles between cfDNA and tumor biopsies reveal discordant results in 23.8% of cases [34]. Given the high concordance (approximately 80%) of genomic mutation profiles between CTCs and tumor biopsies [35], using CTCs as a surrogate target to investigate EGFR mutation profiles shows promise for patients with NSCLC. Nevertheless, even with CTCs, only about 69% of NSCLC patients exhibit concordant EGFR mutation profiles between CTCs and primary tumors at the molecular level. [36] Furthermore, the low concordance (approximately 36% to 50%) between genomic and proteomic profiles suggests limited reliability of molecular-based EGFR CDx for profiling EGFR mutations in either cfDNA or CTCs [37]. Consequently, profiling EGFR mutations via proteomic methods may be more straightforward than current molecular-based approaches.

In this study, we first proposed an approach for investigating EGFR mutations at the proteomic level using flow cytometry. Given its high throughput, sensitivity, and flexibility, flow cytometry is well suited for detecting CTCs in peripheral blood [24]. Current flow cytometry–based CTC detection methods can identify CTCs in various solid malignancies [38]. By combining fluorescent imaging with flow cytometry, researchers can study the morphological changes and intracellular dynamics of certain targets in CTCs [39]. Nevertheless, the currently available protocols primarily focus on detecting the presence and distribution of target proteins instead of protein mutations. In this study, we used immunostaining with flow cytometry to detect a specific EGFR mutation in CTCs (L858R). This method extends flow cytometry-based EGFR detection to cover mutant EGFR and can be expanded to detect other EGFR mutations (e.g., exon 19 deletion, T790M, and G719X) or other TKI targets (e.g., KRAS, MET, ALK, and PD-L1) by including specific antibodies [40,41].

Beyond demonstrating the capability of flow cytometry for direct EGFR mutation detection, this study introduces a novel application to profile EGFR mutation in protein level by flow cytometry. Given that proteomic profiling does not always fully concord with molecular profiling [42], utilizing the proteomic profile of EGFR mutations for assessing clinical responses to TKIs is theoretically a more straightforward approach than relying solely on molecular profiling. Furthermore, by correlating clinical responses to EGFR-TKIs with this proteomic profiling, several promising biomarkers—such as HSPB1 and FGA—have been identified to support the assessment of clinical responses to EGFR-TKIs due to their involvement in EGFR-TKI resistance [43,44]. Nevertheless, a comprehensive evaluation of this proteomic profiling approach and its concordance with clinical responses to EGFR-TKIs remains to be fully conducted. Additionally, similar to molecular profiling, significant tumor heterogeneity presents a considerable risk of confounding targeted therapy assessment due to sampling bias, as documented in cholangiocarcinoma [45,46]. To address these limitations, we propose a novel strategy for profiling EGFR mutations at the protein level by directly detecting mutant EGFR protein on CTCs, which may effectively overcome the challenges posed by tumor heterogeneity.

In our real-world evaluation of the feasibility of the in-house method for detecting EGFR^L858R^-bearing CTCs, we observed a lower negative agreement rate (71%) compared to the positive agreement rate (100%). Specifically, all samples from patients positive by the PCR-based method were also detected as positive by our in-house method, whereas 29% of samples from patients negative by the PCR-based method tested positive with our method. According to the literature, the PCR-based method exhibits a false-positive rate of less than 0.02% for EGFR^L858R^ mutation screening, but its false-negative rate is approximately 11.3% [14]. This discrepancy may stem from tumor heterogeneity at the cellular or genetic level, which can significantly undermine the reliability of PCR-based screening results [47,48,49]. The key objective of our in-house method is to directly detect CTCs harboring a specific phenotype—in this case, the EGFR^L858R^ mutation—and to evaluate the feasibility of applying targeted therapies based on the presence of this phenotype. This approach may provide a more direct and clinically relevant assessment for therapeutic decision-making.

Despite conceptually demonstrating the applicability of detecting EGFR mutations through proteomic evaluation of CTCs, this study has several limitations that restrict the broader application of our in-house method. These limitations include a small sample size and the absence of a correlation between the counts of EGFR^L858R^-bearing CTCs and clinical responses to EGFR-TKIs. Moreover, as patients with wild-type NSCLC are not eligible for open-label use of EGFR-TKIs, we could not investigate the reliability of our in-house method in wild-type disease [50]. Furthermore, the high cost of NGS for solid tumors, coupled with the lack of coverage from the National Health Insurance in Taiwan, has historically limited the number of NSCLC patients undergoing EGFR mutation profiling by NGS, thereby restricting concordant comparisons with our in-house method. To address these limitations, improving the recovery of CTC detection is essential. Additionally, a prospective study examining the correlation between EGFR mutation profiles obtained from our in-house method and clinical response to EGFR-TKIs is warranted. With the coverage of NGS for targeted therapy in solid tumors by the National Health Insurance in Taiwan, future prospective studies can facilitate a more comprehensive concordant investigation between NGS and our in-house method.

## 5. Conclusions

In this study, we developed a flow cytometry–based method to detect CTCs carrying the EGFR^L858R^ mutation in peripheral blood samples from patients with NSCLC. This method allows direct identification of receptor tyrosine kinase mutations at the protein level and supports molecular testing of EGFR to help identify NSCLC patients who may benefit from EGFR-TKI therapy. However, as this is a preliminary study, the assay’s specificity remains limited, and its clinical performance has not yet been fully validated. Further optimization and comprehensive validation in larger patient cohorts are warranted to confirm the assay’s clinical utility.

## Figures and Tables

**Table 1:** Reagents utilized in this study.

Name	Manufacture
For cell culture	
EMEM	Thermo-Fisher, Waltham, MA, USA
Fetal bovine serum	Thermo-Fisher
Ham’s F-12K (Kaighn’s) Medium	Thermo-Fisher
Penicillin/Streptomycin	Thermo-Fisher
RPMI1640	Thermo-Fisher
TrypLE select	Thermo-Fisher
For Immunostaining	
Cell staining buffer	BioLegend, San Diego, CA, USA
Foxp3 Transcription Factor Staining Buffer Set	eBioscience, San Diego, CA, USA
For Spike-in assay	
RBC lysis buffer	Biolegend

Abbreviation: EMEM, Eagle’s minimum essential medium; RBC, red blood cell; RPMI1640, Roswell Park Memorial Institute medium 1640.

**Table 2:** Antibodies utilized in this study.

Target	Clone	Host	Fluorophore	Manufacture	Catalogue No.	Dilution Factor
	Surface markers					
CD45	J33	Mouse	ECD	Beckman Coulter, Brea, CA, USA	A07784	1:10
EGFR^L858R^	43B2	Rabbit	PE	Cell signaling Technology, Denvers, MA, USA	64716S	1:12.5
CD326 (EpCAM)	9C4	Mouse	BV510	BioLegend	324236	1:10
	Intracellular markers					
Cytokeratin 7/8	CAM5.2	Mouse	FITC	BD Pharmingen, Franklin Lakes, NJ, USA	347653	1:20
Cytokeratin 14/15/16/19	KA4	Mouse	AF647	BD Pharmingen	563648	1:80
Vimentin	EPR3776	Rabbit	AF405	Abcam, Cambridge, UK	ab210152	1:125
	Isotype control					
Rabbit mAb IgG XP^®^ Isotype Control	DA1E	Rabbit	PE	Cell Signaling Technology	5742S	1:50

Abbreviation: AF405, Alexa Fluor 405; AF647, Alexa Fluor 647; AF700, Alexa Fluor 700; BV510, Brilliant violet 510; Cy7, Cyanine 7; ECD, Electron coupled dye; FITC, Fluorescein isothiocyanate; PE, Phycoerythrin.

**Table 3:** Participant demographics.

	NSCLC *N* = 21	HC *N* = 10	*p*
Male, N (%)	8 (38%)	6 (60%)	
Median age, year	66 (46–86)	61.5 (50–74)	0.06
Stage, N (%)			
IIIb	1 (5%)		
IV	20 (95%)		
EGFR mutation, N (%)			
Wildtype	8 (38%)		
L858R	7 (33%)		
Other Mutation	6 (29%)		

Abbreviation: HC, cancer-naïve donors; NSCLC, non-small-cell lung cancer.

**Table 4:** Agreement of EGFR^L858R^ detection between PCR-based profiling and in-house CTC-based assay.

Agreement Type	Subject Number (*N*)	Agreement Percentage (%)	Confidence Interval
Overall agreement (CTC^+^/PCR^+^ & CTC^−^/PCR^−^)	17	81	0.6000–0.9233
Positive agreement (CTC^+^/PCR^+^)	7	100	0.6457–1.0000
Negative agreement (CTC^−^/PCR^−^)	10	71	0.4535–1.0000
Disagreement (CTC^+^/PCR^−^ & CTC^−^/PCR^+^)	4	19	
Total	21	100	

PCR-based EGFR^L858R^ detection is based on intratumor EGFR mutation profiling performed by Cobas^®^ EGFR Mutation Test v2. CTC-based EGFR^L858R^ detection is based on CTC EGFR mutation profiling performed by an in-house assay. Confidence interval was calculated by the hybrid Wilson/Brown method.

## Data Availability

The datasets generated during and/or analyzed during the current study are available from the corresponding author on reasonable request.

## Supplementary Materials

The following supporting information can be downloaded at: https://www.mdpi.com/article/10.3390/diseases13120406/s1, Figure S1: Titration of antibodies applied in this study; Figure S2: Fluorescence minus one assay of applied antibody.

## Abbreviations

The following abbreviations are used in this manuscript:

cfDNA	Cell-free DNA
CDx	Companion diagnostics
CNP	Cancer-naïve participant
CTC	Circulating tumor cell
EGFR	Epidermal growth factor receptor
NGS	Next-generation sequencing
NSCLC	Non-small-cell lung cancer
PCR	Polymerase chain reaction
TKI	Tyrosine kinase inhibitor
