# Supplementary material for: Establishment of a Flow Cytometry Protocol for Binarily Detecting Circulating Tumor Cells with EGFR Mutation
Source: Diseases. 2025 Dec 17;13(12):406. doi: 10.3390/diseases13120406 (PMC12731806; doi:10.3390/diseases13120406)
Supplement: Supplementary file 1 [file diseases-13-00406-s001.zip › diseases-3841085-supplementary.pdf]

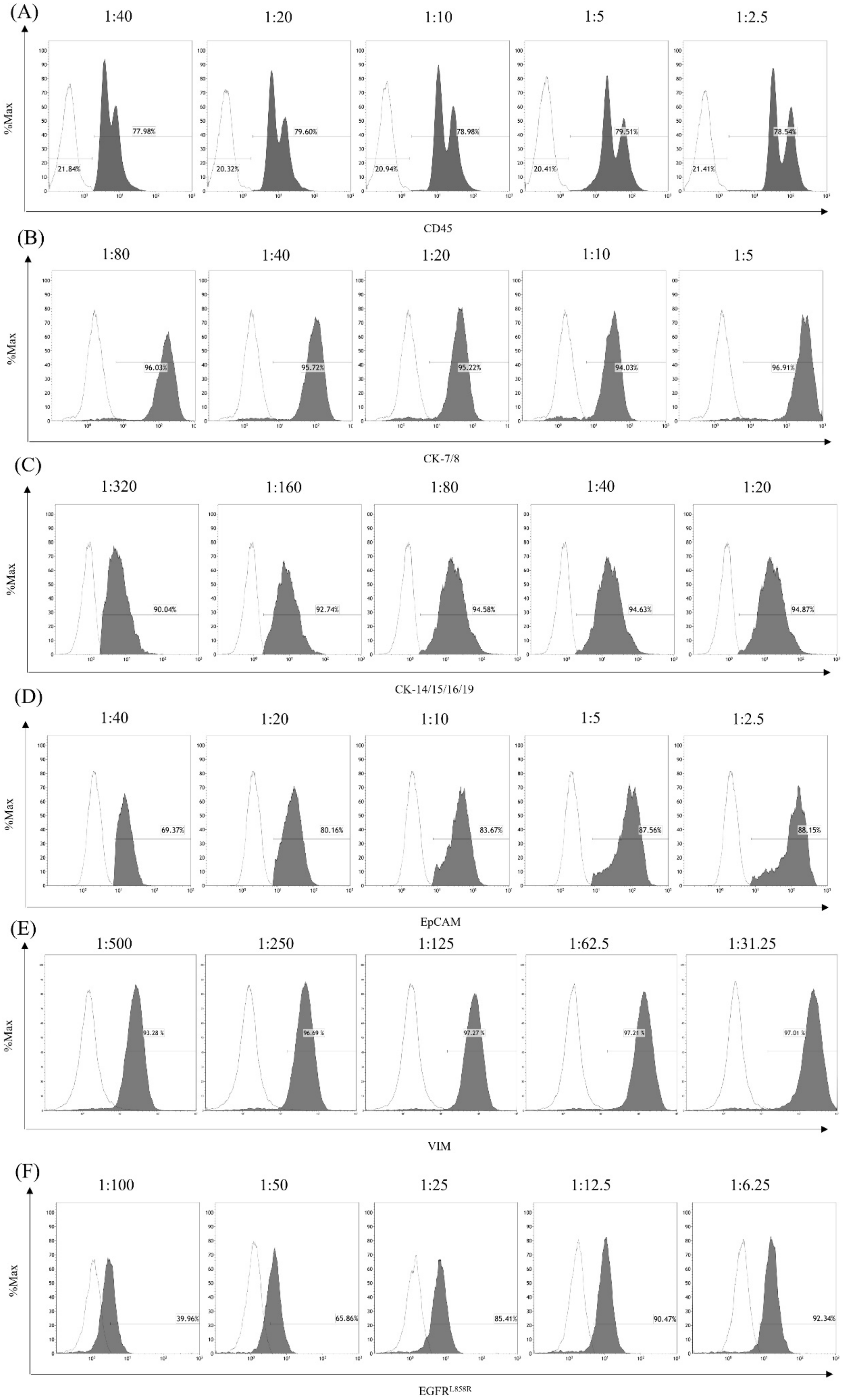
(A)
1:40
1:20
1:10
1:5
1:2.5
%Max
21.84%
77.98%
20.32%
79.60%
20.94%
78.98%
20.41%
79.51%
21.41%
78.54%
CD45
(B)
1:80
1:40
1:20
1:10
1:5
%Max
96.03%
95.72%
95.22%
94.03%
96.91%
CK-7/8
(C)
1:320
1:160
1:80
1:40
1:20
%Max
90.04%
92.74%
94.58%
94.63%
94.87%
CK-14/15/16/19
(D)
1:40
1:20
1:10
1:5
1:2.5
%Max
69.37%
80.16%
83.67%
87.56%
88.15%
EpCAM
(E)
1:500
1:250
1:125
1:62.5
1:31.25
%Max
93.28 %
96.69 %
97.27 %
97.21 %
97.01 %
VIM
(F)
1:100
1:50
1:25
1:12.5
1:6.25
%Max
39.96%
65.86%
85.41%
90.47%
92.34%
EGFRL858R

Supplementary Figure S1. Titration of antibodies applied in this study. Cells expressed desired antigens were stained with targeted antibodies with a series of dilution, followed by analyzing mean fluorescence intensity (MFI) using a flow cytometer. The optimal dilution factor of each antibody was decided by calculating the staining index. Cells applied here were (**A**) peripheal blood mononuclear cells, (**B, D, E, & F**) NCI-H1975 cells, (**C**) SW480 cells.

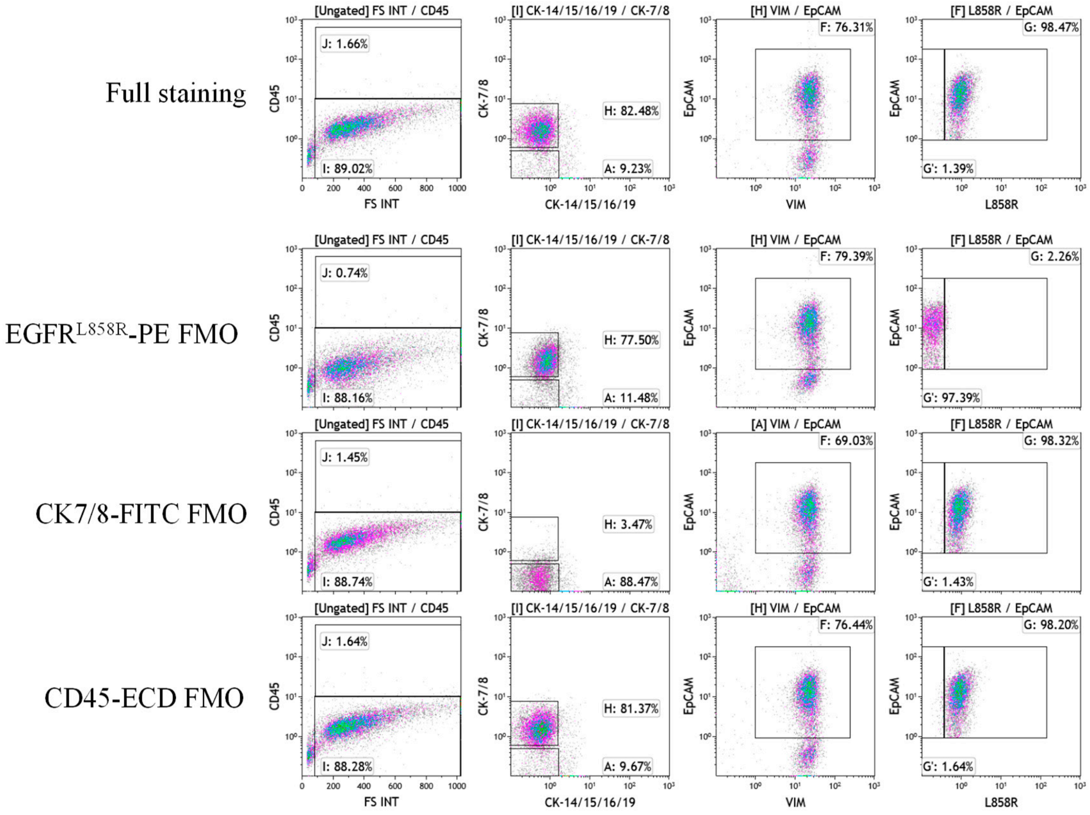

Supplementary Figure S2. Fluorescence minus one assay of applied antibody.

H1975 cells were divided into four aliquots: one for staining with all corresponding antibodies (full staining), one for full staining without EGFR^{L858R}-PE (EGFR^{L858R}-PE FMO), one for full staining without CK7/8-FITC, and one for full staining without CD45-ECD. Following staining, the fluorescence profiles of the cells were analyzed using flow cytometry.
